# Review of chaos in hair-cell dynamics

**DOI:** 10.3389/fneur.2024.1444617

**Published:** 2024-07-10

**Authors:** Justin Faber, Dolores Bozovic

**Affiliations:** ^1^Department of Physics and Astronomy, University of California, Los Angeles, Los Angeles, CA, United States; ^2^California NanoSystems Institute, University of California, Los Angeles, Los Angeles, CA, United States

**Keywords:** chaos, hair cell, signal detection, hearing, vestibular system, nonlinear dynamics, information theory, Hopf bifurcation

## Abstract

The remarkable signal-detection capabilities of the auditory and vestibular systems have been studied for decades. Much of the conceptual framework that arose from this research has suggested that these sensory systems rest on the verge of instability, near a Hopf bifurcation, in order to explain the detection specifications. However, this paradigm contains several unresolved issues. Critical systems are not robust to stochastic fluctuations or imprecise tuning of the system parameters. Further, a system poised at criticality exhibits a phenomenon known in dynamical systems theory as *critical slowing down*, where the response time diverges as the system approaches the critical point. An alternative description of these sensory systems is based on the notion of chaotic dynamics, where the instabilities inherent to the dynamics produce high temporal acuity and sensitivity to weak signals, even in the presence of noise. This alternative description resolves the issues that arise in the criticality picture. We review the conceptual framework and experimental evidence that supports the use of chaos for signal detection by these systems, and propose future validation experiments.

## 1 Introduction

The ability of the auditory system to respond to sound waves that elicit only angstroms of mechanical displacement in the sensory cells has been a subject of research for many decades (1–5). Experiments performed on many scales, from molecular studies to *in vivo* measurements, have revealed the presence of multiple energy-consuming mechanisms that amplify the responsiveness to weak signals, mediate adaptation on different timescales, maintain concentration gradients, and generally provide active feedback dynamics (6–9). While the specific biophysical mechanisms of amplification may vary across the species, the presence of some active process seems to be ubiquitous, and the resulting nanoscale sensitivity prevalent. A common feature of all auditory end organs is that the sensory cells are immersed in an aqueous environment, and hence their stereociliary bundles are subject to stochastic noise and the resulting viscous damping. Thus, the sensitivity of detection must be considered not only in terms of amplification of weak signals, but in the context of extracting signals from equal or higher levels of noise.

Nonlinear response has likewise been demonstrated in the dynamics of individual hair cells (10–12), semi-intact end organs, and otoacoustic emissions of live animals (13, 14). The nonlinearity is closely linked to and dependent upon the active process, as it vanishes with metabolic disruption. Further, it is dominant at weak signals, indicative of an essential nonlinearity. These empirical data have motivated theoretical models based on nonlinear dynamics, which have proposed that the hair cell can be described as an active oscillator positioned near a supercritical Hopf bifurcation (15–17). Proximity to a critical point has been shown to capture the nonlinearity, amplification, frequency selectivity, and ability to exhibit autonomous oscillation (18–21). In the vicinity of this bifurcation, the response of the system is generic, not dependent on the microscopic mechanisms governing the active oscillator. Thus, the behavior of complex numerical models near criticality can be captured by the normal form equation for the Hopf bifurcation (22). Consistent with a large body of experimental evidence, this theoretical framework provides a powerful paradigm for describing the dynamics of the auditory system, with the hair cell as the key element.

While criticality provides many advantages, several limitations arise in the full characterization of the response of this dynamical system. Firstly, criticality is only optimal in a noiseless, deterministic system. In this regime, the bifurcation point provides maximal amplification, and as the system crosses into the oscillatory, limit-cycle regime, its sensitivity is degraded. In the presence of noise, criticality is removed, in the sense that a singular point dividing the quiescent and oscillatory regimes is replaced by a gradual transition (23). The regime around the critical point still exhibits many features of criticality, such as amplification and nonlinear response. However, in the presence of noise, it was shown that a stable limit cycle, rather than proximity to bifurcation, provides maximal sensitivity (24).

Secondly, a noiseless system poised exactly at the supercritical Hopf bifurcation would exhibit a phenomenon known as *critical slowing down* (25), where infinitely long transient times of the response arrise from the loss of local stability. Thus, when the system is maximally sensitive, it is also maximally slow. For a model of the auditory system, this limitation is highly significant, as experimental studies have shown that many phenomena, such as localization of sound in space, require temporal acuity that reaches 10 microseconds (26, 27). In the presence of noise, critical slowing down is reduced, but so is the sensitivity. The inherent tradeoff between the sensitivity and speed of response persists, posing a highly undesirable tradeoff for auditory detection.

Nonlinear dynamics theory provides another class of systems, that does not rely on criticality to achieve sensitivity. Chaotic attractors have received much attention in mathematics and physics literature, as they provide intricate fractal patterns in their trajectories, exhibit universal phenomena, and model the behaviors of systems as far ranging as lasers, transistors, mercury films, and others (25). Of relevance to biology are several key features of chaotic systems. First, they require only three degrees of freedom to arise in a dynamical system. As biology typically involves a multiplicity of interacting active processes, one expects many more dimensions than three to characterize its dynamics, making the occurrence of chaos easily feasible. Secondly, chaos reconciles sensitivity of detection with temporal resolution. In fact, the very definition of chaos relies on this feature: the trajectories of a chaotic system diverge exponentially as a result of infinitesimally small perturbation. This divergence of trajectories thus reflects the sensitivity of response and imposes no tradeoff with its speed.

Furthermore, while chaotic dynamics can be obscured by the presence of noise, their key features are not removed. On the contrary, certain dynamical systems that show stable limit cycles when noiseless can be rendered chaotic by the introduction of stochastic fluctuations (28). Specifically, the theoretical model for the Hopf bifurcation, in its most general form, exhibits a chaotic regime in the presence of noise (29). This regime would enable the system to exhibit its requisite sensitivity as well as maintain rapid response, achieving these features in the presence of noise levels comparable to those expected in biological tissue.

## 2 Chaos in hair-bundle dynamics

Most theoretical and experimental studies of chaos in the auditory and vestibular systems have focused on the sensory elements - individual hair cells. As these sensory cells exhibit all of the key signatures of the active, nonlinear response that characterizes hearing, they provide a natural starting point for the analysis of underlying dynamics. However, defining features of chaos have traditionally been explored in theoretical models, mostly deterministic in nature, as they can be difficult to identify even in numerical simulations that introduce stochastic elements. Hence, proving the presence of chaos in experimental systems poses a challenge, due to the presence of thermal fluctuations and limitations in measurement precision and duration (30, 31). For instance, the standard metric for quantifying chaos in a numerical model is the Lyapunov exponent, which quantifies the divergence rate of neighboring trajectories and estimates how rapidly uncertainties in the present state of a system diverge. Extracting the Lyapunov exponent from experimental data can prove unreliable, as the presence of noise limits measurement precision, thereby obscuring the degree of separation of two phase-space trajectories.

Despite the challenges, time-series analysis techniques have been developed in applied mathematics, which can identify the presence of chaos in experimental recordings and even quantify its degree. Specifically, a fruitful approach for the analysis of experimental data is to identify a transition to the chaotic regime, usually through variation of an experimental parameter. Transitions to chaos tend to arise from one of several well-known routes. The period-doubling route has been identified in stimulated squid giant axons (32), while the quasiperiodic route has been shown to occur in the intervals between heartbeats, at the onset of cardiac fibrillation (33, 34).

Identifying these transitions involves taking “snapshots” of the system through the use of Poincaré maps. For example, in the case of heartbeats, one may capture the time interval between two subsequent heartbeats (*I*_*n*_) and form a scatter plot against each interval that follows (*I*_*n*+1_). Regular heartbeats would correspond to a single cluster of points in this plane. Multiple clusters of points would indicate occasional skipping of beats, or some form of mode-locking behavior. Alternatively, a ring-like structure in this Poincaré map would indicate the presence of two incommensurate frequencies (quasiperiodicity) in the dynamics. Quasiperiodic dynamics correspond to trajectories on the surface of a 2-torus, where the ringlike structure represents a cross-section of the torus. A common route to chaos in dynamical systems theory is torus breakdown, where the surface of the torus loses smoothness, giving rise to chaotic dynamics (35).

**Figure 1:**
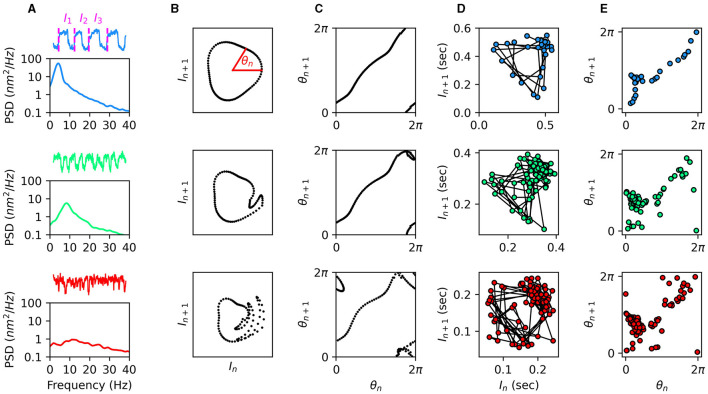
**(A)** Traces and power spectral density of spontaneous hair-bundle oscillations under various calcium concentrations of the endolymph. From top to bottom: 100 μ*M* (low calcium), 250 μ*M* (natural calcium), and 325 μ*M* (high calcium). Oscillation intervals are illustrated in the top trace. **(B)** Illustrations of the torus-breakdown transition to chaos from top to bottom. **(C)** Circle maps corresponding to the Poincaré maps in **(B)**. The 1-to-1 relationship in the top panel is indicative of non-chaotic dynamics, while the other two panels are indicative of chaos. **(D)** Experimental measurements of oscillation intervals from a hair bundle with the three calcium concentrations in **(A)** during an off-resonance stimulus. **(E)** Circle maps corresponding to the Poincaré maps in **(D)**. The 1-to-1 relationship in the top panel (low calcium) is indicative of non-chaotic dynamics, while the other two panels are indicative of chaos. Data was reproduced from (29) with permission.

The approach of utilizing Poincaré maps was performed on spontaneously oscillating hair bundles of the bullfrog sacculus, in order to identify chaos in the dynamics of individual sensory hair cells (36). The calcium concentration and viscosity of the endolymph solution surrounding the hair bundles were varied, as these have been shown to influence the regularity of oscillations (37). In Figure 1A, we show traces of hair-bundle spontaneous oscillations for three choices of endolymph calcium concentration. For low values of the calcium concentration, the hair bundles exhibit regular oscillations and a tall, narrow peak in the power spectrum. However, as the calcium concentration is increased, the oscillations become more irregular and more chaotic (29).

An external, sinusoidal stimulus was then introduced to the system. Variation in the amplitude of this stimulus was used to control the system, with large amplitudes causing entrainment of the hair bundle and resulting in limit-cycle dynamics. In Figure 1B, we illustrate the anticipated Poincaré maps for a quasiperiodic transition to chaos via torus breakdown. The smoothness of the torus can be tested using circle maps, where the angle each point makes with the *I*_*n*_-axis is plotted against the angle of the point that follows. Smoothness can be identified if there is a 1-to-1 relationship in the circle map, such that the points follow an invertible, monotonic function (Figure 1C) (35). The experimental data shown in Figures 1D, E indicates that for low calcium concentrations, the hair bundle exhibits limit-cycle dynamics. However, for natural and high calcium concentrations, the system exhibits chaotic dynamics.

Though the Poincaré maps are useful for identifying chaos, they provide no information about the degree of chaoticity. The Kolmogorov entropy provides a useful metric for quantifying chaos, with larger values corresponding to more limited predictability (38, 39). Closely related to the Lyapunov exponents of a dynamical system, this information-theoretic metric quantifies how rapidly phase-space information is lost with time, due to the expansion of uncertainties (40). While Lyapunov exponents track the divergence of distances between neighboring trajectories, the Kolmogorov entropy tracks expansion rates of volumes confining local neighborhoods of the phase space. Though its algorithmic implementation is more complex than that of the Lyapunov exponent, measuring the Kolmogorov entropy of time-series data does not rely on extremely high measurement precision. Instead, the phase space is partitioned into discrete bins, and the flow of trajectories through these bins is used to calculate the rate at which the probability distribution of local trajectories spreads out. This metric was implemented to quantify the level of chaos in hair-bundle dynamics. As expected, increasing the calcium concentration of the endolymph increased the Kolmogorov entropy (29).

## 3 Signal detection by chaotic oscillators

Exploiting the instabilities of chaotic systems in the design of sensitive signal detectors was proposed decades ago (41, 42). This idea was likewise tested on a detailed biophysical model of hair-cell response, which was shown to be most sensitive to a step-like stimulus when the system was poised in the chaotic regime (28). Subsequent theoretical studies further demonstrated that a simple dynamical systems model of a hair cell, based on a generalized form of the Hopf bifurcation, greatly benefits from the chaotic regime, providing signal detection that is more robust to thermal noise than the equivalent system poised at criticality (23). To test these theoretical predictions on live and biologically functional hair cells, the level of chaos was first measured from long recordings of spontaneous hair-bundle oscillations. At the end of each recording, the hair bundle was stimulated with an external signal, and its responsiveness was measured. Thus, the level of chaos observed in the innate dynamics of a hair bundle could be directly related to its responsiveness to an external mechanical stimulus. The level of chaos was then adjusted though variation of the calcium concentration or viscosity of the endolymph, and the identical experimental protocol was repeated (29).

**Figure 2:**
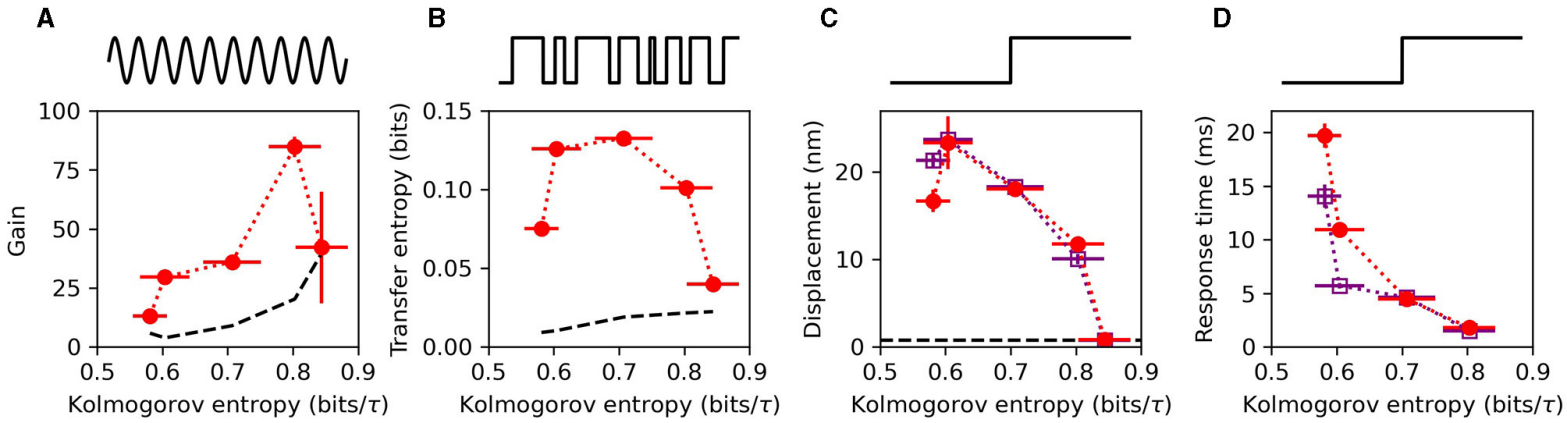
**(A)** Phase-locked amplitude gain for weak sinusoidal stimulus, presented at the characteristic frequency of the bundle. **(B)** Transfer entropy from burst-noise stimulus to the hair bundle response. **(C)** mean, steady-state displacement induced on the hair bundle from a step stimulus, averaged over ~200 steps. **(D)** Response time to the step stimulus, characterized by fitting the mean response to an exponential function and extracting the decay time. The black, dashed curves in **(A–C)** represent the noise floors associated with each metric. In **(C, D)**, the red circles, and purple squares represent stimulus steps in the channel-opening and channel-closing direction, respectively. All measurements were performed on the same hair cell. Stimulus waveforms used for each metric are illustrated above each panel. Data was reproduced from (29) with permission.

To characterize the hair-bundle sensitivity, several metrics were used. First, a pure tone was applied, and the amplitude gain was measured at the stimulus frequency (Figure 2A). This metric is appropriate for the frequency-selective detectors of the auditory system. Telegraphic noise (burst noise) was also applied to the bundle (Figure 2B). This allowed for calculation of the transfer entropy, a metric that quantifies how much information the receiver obtains about the stimulus (43). The advantage of this metric is that it makes no assumptions about what components of the signal are relevant, such as the amplitude, frequency, or phase. Instead it directly measures how much information about the stimulus is encoded in the response. Lastly, a step-function stimulus was repeatedly applied to the bundle (Figure 2C), and the mean displacement of the response was calculated. This metric is appropriate for characterizing broadband detection by vestibular systems, as step functions are transient and contain a broad range of frequencies in their Fourier representation. All three of these metrics showed enhanced responsiveness in the chaotic regime and exhibited a single peak as a function of the Kolmogorov entropy. These experimental results are consistent with theoretical predictions, which demonstrated that optimal signal detection occurs in the weakly chaotic regime (23, 29).

The speed of response could also be extracted from these experimental recordings, providing a measure of the temporal acuity of individual hair bundles. Using multiple repetitions of a step-function stimulus, the responses were averaged to reveal an exponentially decaying function. The timescale obtained from exponential fits to the data yielded the response time (Figure 2D). For all of the hair cells measured, the speed of response increased monotonically with increasing levels of chaos. These results are consistent with analytic calculations of the phenomenological Hopf model for hair cells (44).

The monotonic increase in temporal acuity with increasing level of chaos, combined with the optimum observed in the sensitivity metrics, suggests that the preferred level of chaos for signal detection depends on the specific application and requirements for the detector. For example, a recent theoretical study, which considered many detection metrics, proposed that weakly chaotic oscillators perform best as auditory detectors, while strongly chaotic oscillators are best suited for vestibular detection (23).

## 4 Mechanically-coupled chaotic oscillators

According to a theoretical study, the only detection metric that is degraded by the presence of chaos is the frequency selectivity of the system, as quantified by the quality factor of the response (23). This is to be expected of chaotic oscillators, as they tend to exhibit broad ranges of frequencies in their Fourier representation and are susceptible to entrainment within this frequency range. While this poses no tradeoffs for the broadband detectors of vestibular systems, it raises the question of how auditory systems achieve their frequency selectivity if they are comprised of chaotic oscillators. One possible resolution to the issue of frequency selectivity was proposed in a recent study (45), which demonstrated that mechanically coupled, active oscillators are more likely to synchronize when they are individually chaotic. This study also confirmed that the increase in synchronization susceptibility was accompanied by an increase in frequency selectivity of the full, coupled system. Though the individual detectors were not frequency selective, when many synchronize their autonomous motion, they form a sharply-tuned, frequency-selective system. Mechanical coupling between hair cells is present in both auditory and vestibular systems, and the strength and extent of this coupling varies between species and depends on the function of the sensory system (46). This raises the possibility that chaotic dynamics can persist in large arrays of coupled hair cells, and that specific morphology of the overlying structures fine-tunes the level of chaos and the emergent detection characteristics of the full system.

## 5 Discussion

Our theoretical studies have prioritized the inclusion of stochastic noise in measurements of sensitivity, as it drastically affects the performance of signal detectors and can qualitatively alter the conclusions as to which regime in parameter space yields optimal detection. Furthermore, we propose that both theoretical and experimental studies can benefit from using information theoretic metrics to characterize the response of a hair bundle. These metrics allow one to assess how external stimuli impact the dynamics of a cell, beyond the effects on its mechanical compliance. For example, transfer entropy provides a particularly useful measure for characterizing the sensitivity of hair cells, as it caries no assumptions of which signal features are neurologically meaningful.

We note that the experimental measurements of chaotic dynamics have thus far been performed only on hair cells of the amphibian sacculus, a primarily vestibular end organ. Our theoretical studies, however, suggest that the benefits entailed in chaotic dynamics may be far more generic and hence applicable to other systems as well. Vertebrate species indeed exhibit a diverse range of sensory organs, specialized to perform both auditory and vestibular tasks. While the morphology of their macroscopic structures varies widely, these systems universally rely on active hair bundle motility, somatic electromotility, or a combination thereof (46). Prior work has demonstrated that nonlinear dynamic models of active hair cells are very versatile and can describe a broad range of sensory specializations (47, 48).

We hence propose that the auditory system exploits the instabilities inherent to chaotic dynamics in order to achieve its remarkable sensitivity, temporal acuity, and robustness to noise. Future experimental studies will aim to test these theoretical predictions by exploring whether chaotic dynamics can arise in auditory end organs under natural conditions. One experimental study found signatures of chaos in the spontaneous otoacoustic emissions of humans (49), raising the possibility that the auditory system exhibits chaoticity *in vivo*.

## Author contributions

JF: Writing – original draft, Writing – review & editing. DB: Writing – original draft, Writing – review & editing.
